# Gefäßkomplikation bei Beckenringstabilisierung: Fallbericht einer iatrogenen Verletzung der Arteria glutea superior

**DOI:** 10.1007/s00113-026-01725-8

**Published:** 2026-06-30

**Authors:** Anna L. Schiltenwolf, Christof K. Audretsch, Tina Histing, Steven C. Herath, Markus A. Küper

**Affiliations:** 1 Unfall- und Wiederherstellungschirurgie, BG Unfallklinik Tübingen, Schnarrenbergstraße 95, 72076 Tübingen, Deutschland; 2 Universitätsklinikum Tübingen, Tübingen, Deutschland

**Keywords:** Beckenringfraktur, A. glutea superior, Iatrogene Gefäßverletzung, Pseudoaneurysma, Fixation mit Sakroiliakalschrauben, Pelvic ring fracture, Superior gluteal artery, Iatrogenic vascular injury, Pseudoaneurysm, Sacroiliac screw fixation

## Abstract

Die A. glutea superior ist bei Beckenverletzungen und -operationen besonders gefährdet. Wir berichten über einen 74-jährigen Patienten mit instabiler Beckenringfraktur, initial versorgt mit Fixateur externe und bilateralen Sakroiliakalschrauben (SI). Postoperativ zeigten persistierend erhöhte Entzündungswerte und ein persistierend niedriger Hämoglobinspiegel Hinweise auf ein am ehesten iatrogen entstandenes Hämatom. Die CT-Diagnostik bestätigte ein 2 × 3 cm großes Pseudoaneurysma der A. glutea superior. Nach Thrombininjektion, Hämatomausräumung und Ligatur konnte eine erfolgreiche Hämostase erreicht werden. Dieser Fall unterstreicht, dass sorgfältige Planung, Kenntnis der Gefäßanatomie und frühzeitige Beachtung von Laborparametern entscheidend zu Prävention und Erkennung von Gefäßverletzungen bei SI-Schrauben-Implantationen sind.

## Abstract (en)

The superior gluteal artery is particularly at risk in pelvic fractures and surgical procedures. We report on a 74-year-old male patient with an unstable pelvic ring fracture, initially treated with an external fixator and bilateral sacroiliac (SI) screws. Postoperatively, persistently elevated inflammatory markers and a persistently low hemoglobin level suggested a hematoma most likely from an iatrogenic cause. Computed tomography (CT) imaging confirmed a 2 × 3 cm pseudoaneurysm of the superior gluteal artery. Hemostasis was successfully achieved through thrombin injection, hematoma evacuation and ligation. This case highlights that careful preoperative planning, detailed knowledge of vascular anatomy and early attention to laboratory parameters are essential for the prevention and detection of vascular injuries during SI screw implantation.

## Einleitung

Beckenfrakturen machen etwa 3 % aller traumatischen Verletzungen aus [3]. Bei instabilen, gering verschobenen Frakturen des hinteren Beckenrings ist die perkutane Fixierung mit Sakroiliakalschrauben (SI-Schrauben) eine etablierte Behandlungsmethode [7]. Die intraoperative Navigation und 3D-Bildgebung erleichtern die präzise Platzierung der Schrauben und verringern das Risiko einer Fehlpositionierung.

Der Eintrittspunkt für den Führungsdraht wird in der Regel im seitlichen Röntgenbild bestimmt. Unter wiederholter Bildgebung in Inlet- und Outlet-Projekt wird der Draht eingebracht, anschließend wird der Draht überbohrt. Dies kann auch durch Navigation unterstützt erfolgen. Schließlich wird die SI-Schraube eingebracht oder eingedreht [5].

Trotz standardisierter Techniken sind iatrogene Verletzungen neurovaskulärer Strukturen weiterhin möglich. Das Risiko kann durch individuelle anatomische Variationen der Beckengefäße, insbesondere in der Iliakalregion, erhöht werden [1, 2, 8].

## Anamnese

**Abb. 1:**
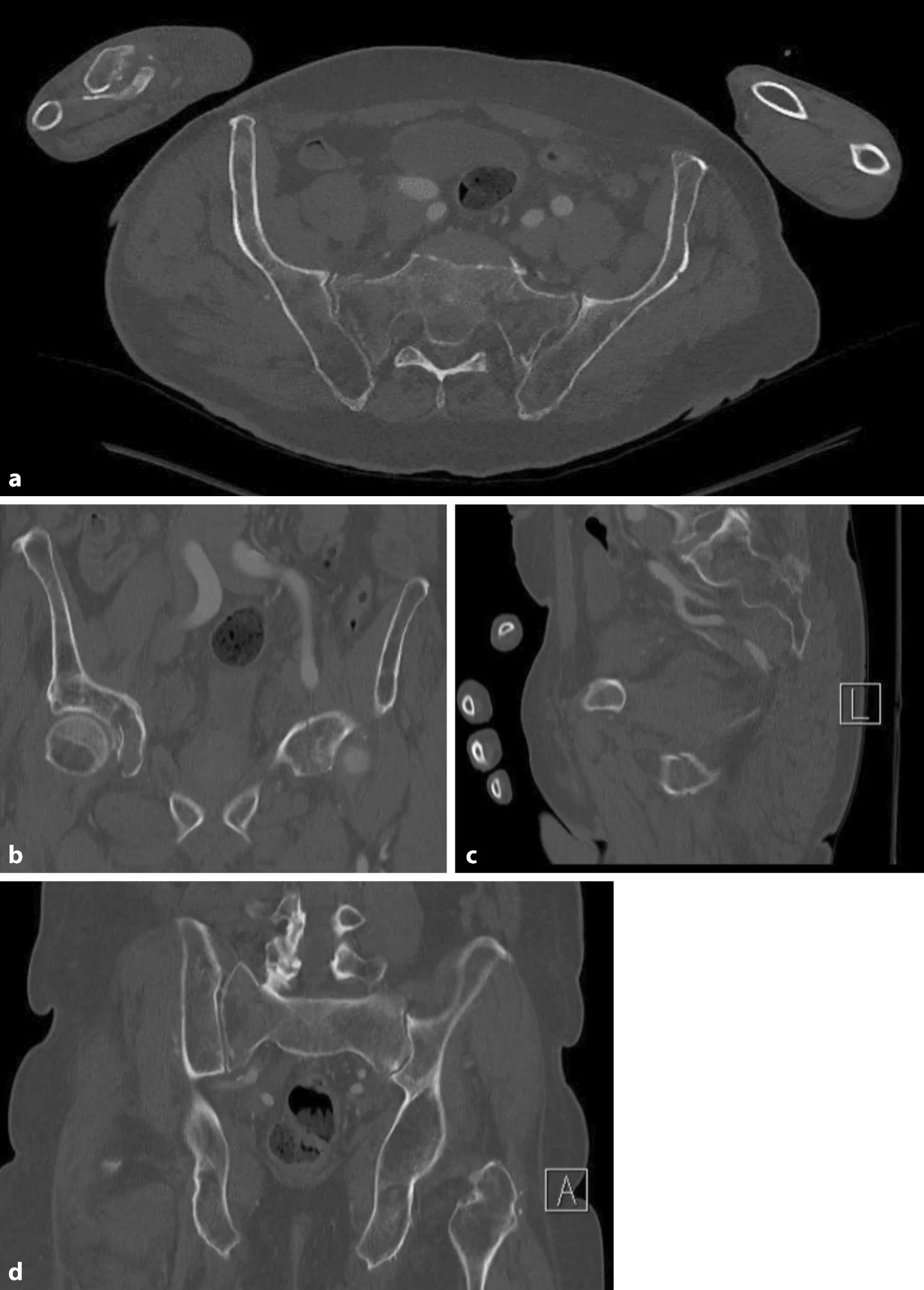
Präoperatives CT der instabilen Beckenringfraktur mit beidseitigen Frakturen des oberen und unteren Schambeinastes sowie H‑förmiger Sakrumfraktur (**a** axial, **b** und **d** koronar, **c** sagittal)

**Abb. 2:**
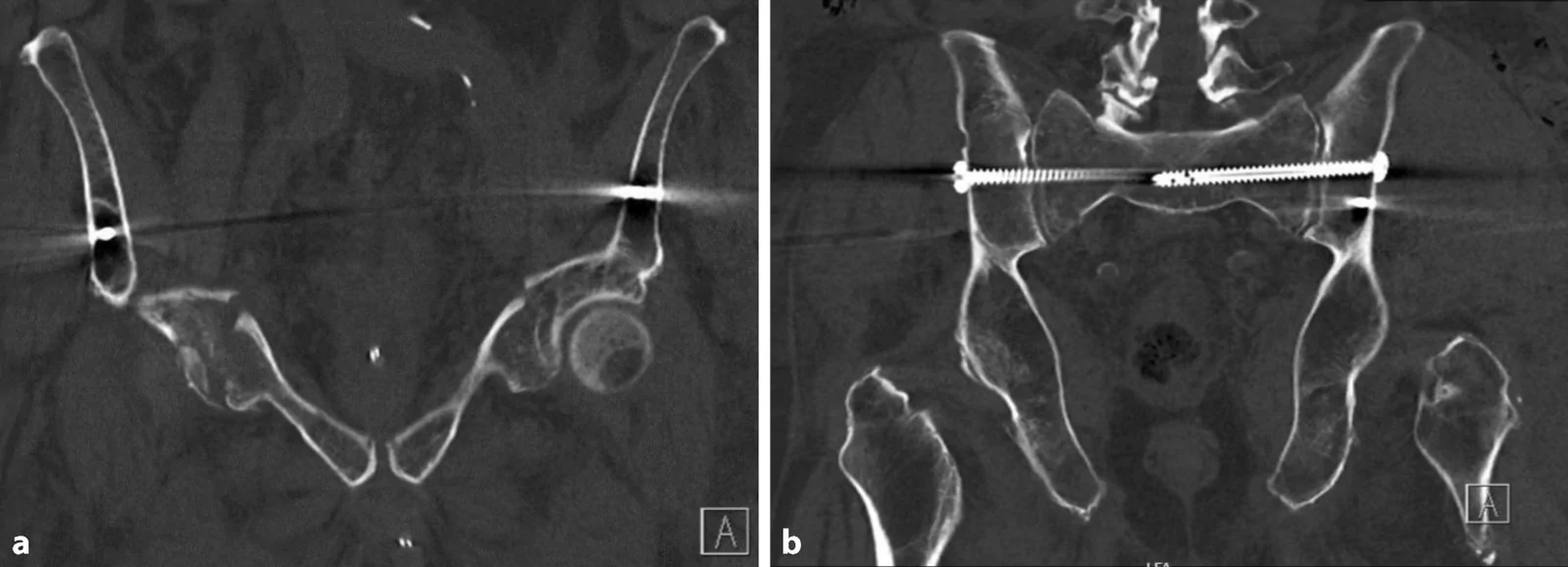
Postoperatives CT mit einliegendem supraacetabulärem Fixateur externe und beidseitig eingebrachten Iliosakralschrauben (**a** und **b** koronar)

Ein 74-jähriger Mann wurde nach einem Motorradunfall bei einer Geschwindigkeit von etwa 50 km/h mit einer instabilen Beckenringfraktur mit beidseitiger Beteiligung der Massa lateralis und des vorderen Beckenrings vorgestellt (Abb. 1). Die primäre Diagnostik umfasste eine klinische Untersuchung, eine eFAST sowie eine kontrastmittelgestützte Ganzkörper-CT („Traumaspirale“). Hierbei ergaben sich keine Hinweise auf eine aktive arterielle Blutung. Die chirurgische Stabilisierung erfolgte mit einem supraacetabulären Fixateur externe und beidseitigen SI-Schrauben (Abb. 2). Intraoperativ zeigte sich entlang des Zugangs zur linken SI-Schraube ein subkutanes Hämatom, das einer Morel-Lavallée-Läsion entspricht und mit einem erheblichen Blutverlust einherging, der die Transfusion von 2 Erythrozytenkonzentraten erforderlich machte. Während der Intensivbehandlung war eine weitere Transfusion erforderlich.

Im Bereich des Zugangs zur linken SI-Schraube trat postoperativ eine Wundsekretion auf, die im weiteren Verlauf sistierte. Der externe Fixateur wurde 2,5 Wochen postoperativ aufgrund einer Lockerung entfernt. Drei Wochen nach der Verletzung wurde der Patient zur Rehabilitation entlassen. Vier Tage später wurde der Patient erneut mit erhöhten Entzündungswerten vorstellig.

## Befund

**Abb. 3:**
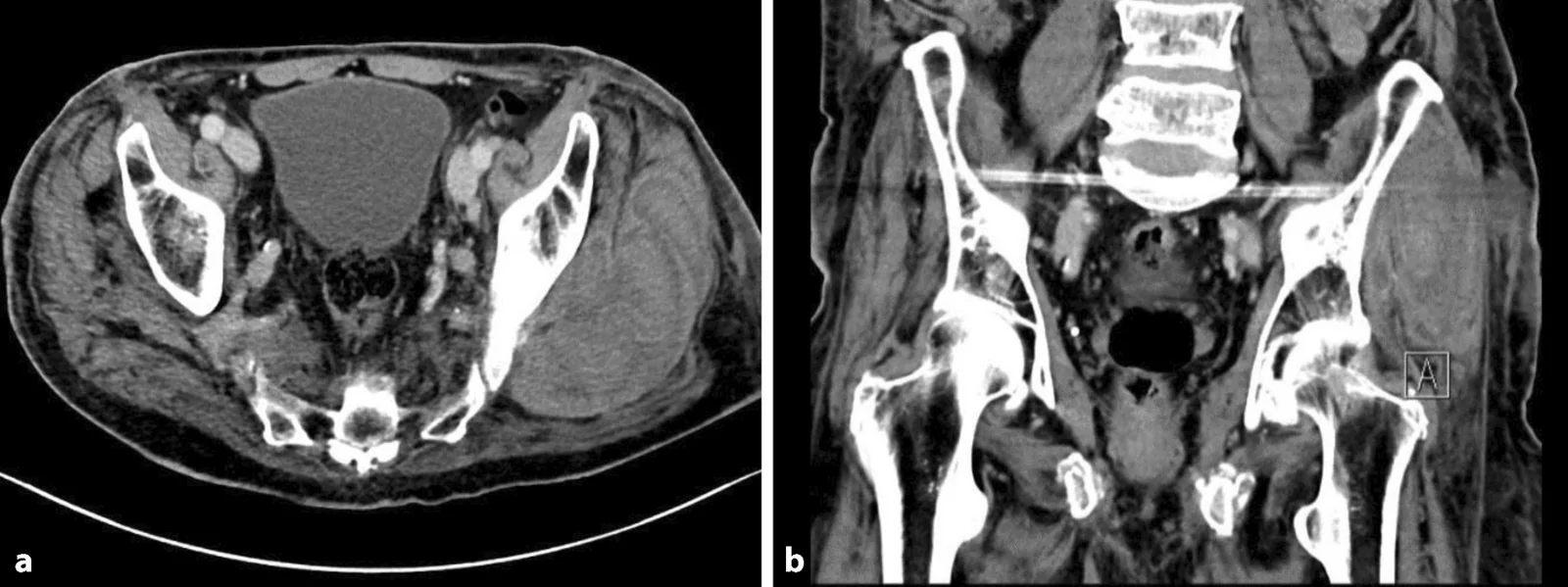
Kontrastmittelgestützte Becken-Bein-CT-Angiographie mit Darstellung eines großen glutealen Hämatoms und umschriebener Kontrastmittelaussparung sowie Nachweis eines Pseudoaneurysmas der A. glutea superior (**a** axial, **b** koronar)

Die Laboruntersuchungen zeigten eine Leukozytose (11.950/µl), erhöhte CRP-Werte (19,97 mg/dl) sowie eine anhaltende normochrome, normozytäre Anämie (Hämoglobin 8,7 g/dl). Klinisch präsentierte sich der Patient stabil und nur mit geringen Schmerzen; eine standardisierte Schmerzerfassung mittels visueller Analogskala (VAS) erfolgte nicht. Die Operationswunden waren trocken und ohne Anzeichen einer Infektion. Die kontrastverstärkte CT zeigte allerdings ein linksseitiges Glutealhämatom, sowie ein 2 × 3 cm großes Pseudoaneurysma der A. glutea superior, wobei eine iatrogene Genese im Rahmen der operativen Versorgung als wahrscheinlich erscheint, eine primär traumatische Ursache im Sinne einer Sickerblutung jedoch nicht sicher ausgeschlossen werden kann (Abb. 3).

## Therapie

Die Erstbehandlung umfasste eine CT-gesteuerte Thrombininjektion, gefolgt von einer chirurgischen Hämatomentfernung und einer Ligatur der A. glutea superior.

## Verlauf

**Abb. 4:**
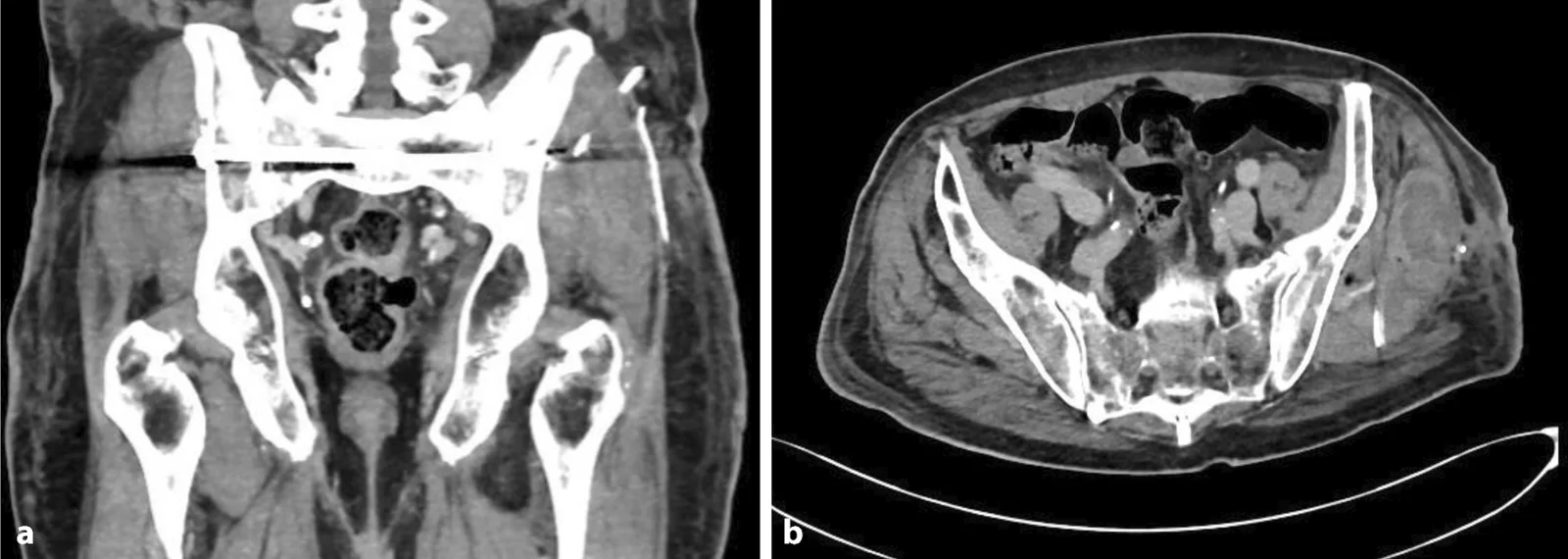
Postoperative kontrastmittelgestützte Becken-Bein-CT-Angiographie ohne Nachweis einer aktiven Blutung im Bereich des ehemaligen Pseudoaneurysmas. Das Hämatom zeigte eine regredierte Größe; einliegende Drainagen (**a** koronar, **b** axial)

Die postoperative Kontrastmittel-CT bestätigte die Blutstillung und die Rückbildung des Hämatoms (Abb. 4). Der Patient konnte unter physiotherapeutischer Anleitung sicher mit einer Gehhilfe mobilisiert werden und wurde 5 Tage später zur Rehabilitation entlassen. Die intraoperativ gewonnenen Abstriche blieben ohne Erregernachweis.

## Diskussion

Der oben beschriebene Fall wirft Fragen zu den Ursachen und Präventionsstrategien iatrogener Gefäßverletzungen im Rahmen der SI-Schrauben-Implantation auf. Die A. glutea superior, ein Ast der A. iliaca interna, verlässt das Becken durch das Foramen ischiadicum majus und teilt sich in einen oberflächlichen und einen tiefen Ast, die die Gesäßmuskeln versorgen. Obwohl iatrogene Verletzungen dieses Gefäßes selten sind (0,6–1,2 %), ist ihre klinische Relevanz hoch. Die Arterie ist sowohl bei traumatischen Beckenverletzungen als auch im Rahmen chirurgischer Eingriffe gefährdet, insbesondere bei der Implantation von SI-Schrauben, da Fehlpositionierungen oder Abweichungen vom sicheren Knochenkorridor Gefäßschäden verursachen können. [1, 4, 6, 9]. Zu den Risikofaktoren zählen anatomische Variationen, überdimensionierte Führungsdrähte und wiederholte Neupositionierungen. Eine präoperative angiographische Bildgebung kann dabei helfen, den Verlauf der vaskularen Strukturen darzustellen, Gefäßanomalien zu erkennen und das Risiko einer iatrogenen Verletzung zu verringern [9].

In unserem Fall lässt sich die Ursache der Gefäßverletzung retrospektiv nicht eindeutig klären. Differenzialdiagnostisch kommen sowohl eine primär traumatische als auch eine im Rahmen der operativen Versorgung entstandene Gefäßläsion in Betracht, wobei Letztere aufgrund des zeitlichen Zusammenhangs als am ehesten wahrscheinlich erscheint. Dies unterstreicht, dass selbst bei standardisierter präoperativer Vorbereitung, intraoperativer Bildgebung und Durchführung durch einen erfahrenen Operateur ein Restrisiko für vaskuläre Komplikationen besteht. Selbst bei Einhaltung der Standardverfahren bleibt ein Restrisiko bestehen. Die Diagnosestellung wurde aufgrund eines zunächst unauffälligen Verlaufs verzögert, was die Bedeutung einer genauen Beobachtung anhaltender oder neu auftretender Symptome nach einer Operation unterstreicht. Auffällig war in unserem Fall das Fehlen ausgeprägter Schmerzen trotz relevanter Raumforderung, was die klinische Diagnosestellung zusätzlich erschwerte. Der vorliegende Fall hat das Bewusstsein für das bestehende Restrisiko vaskulärer Komplikationen geschärft. Bei unklaren erhöhten Entzündungsmarkern nach einer Beckenoperation sollten nach Ausschluss einer Infektion weitere gefäßdiagnostische Maßnahmen in Betracht gezogen werden.

## Schlussfolgerung

Seltene Komplikationen wie Verletzungen der A. glutea superior sollten in die präoperative Risikobewertung einbezogen werden. Eine sorgfältige Planung unter Berücksichtigung der individuellen Gefäßanatomie und atypischer postoperativer Befunde ist für eine frühzeitige Diagnose und Behandlung von entscheidender Bedeutung. Persistierend erhöhte Entzündungsmarker in Kombination mit einer ausbleibenden postoperativen Erholung des Hämoglobinspiegels sollten den Verdacht auf eine Gefäßverletzung lenken.

## Data Availability

Alle dieser Arbeit zugrunde liegenden Daten sind in diesem Artikel enthalten.
